# An Overview of the Role of Genetic factors in Idiopathic Pulmonary Fibrosis: Insights from Epidemiology to Prognosis

**DOI:** 10.7150/ijms.113226

**Published:** 2025-06-12

**Authors:** Jiahao Liu, Zihan Yi, Ting Chen, Yinghua Ying, Yue Hu

**Affiliations:** 1 Key Laboratory of Respiratory Disease of Zhejiang Province, Department of Respiratory and Critical Care Medicine, Second Affiliated Hospital of Zhejiang University School of Medicine, Hangzhou 310009, Zhejiang, China.; 2 Department of Endoscopy Center, The Second Affiliated Hospital, Zhejiang University School of Medicine, Hangzhou 310009, Zhejiang, China.

**Keywords:** genetics, idiopathic pulmonary fibrosis (IPF), MUC5B, surfactant, telomerase

## Abstract

Idiopathic pulmonary fibrosis (IPF), a chronic progressive fibrosing interstitial lung disease with an unclear etiology, is characterized by progressive respiratory impairment and a median survival of 3-5 years. The pathophysiology associated with genetic factors in IPF remains largely unknown, despite the fact that both familial and sporadic IPF exhibit genetic susceptibility. In this review, we comprehensively examine genetic variations associated with the functional roles of mucin 5B (MUC5B), telomerase complex, surfactant proteins, cytokines, signaling pathways, and epigenetic mechanisms. A multifaceted perspective derived from genetic, epidemiological, and clinical studies demonstrates that genetic variations exert differential impacts on the development, progression, and prognosis of IPF. We advocate for the application of genetic knowledge to facilitate the refinement of diagnostic approaches, enhance the assessment of therapeutic strategies and prognostic outcomes, and underscore the significance of personalized therapy for IPF.

## 1. Introduction

Idiopathic pulmonary fibrosis (IPF) is a chronic interstitial lung disease (ILD) that primarily affects adults and is characterized by fibrosis and clinical symptoms such as dyspnea and progressive deterioration of pulmonary function. IPF typically has an unfavorable prognosis with a median survival of approximately three to five years 10. The disease manifests in both sporadic and familial forms, each exhibiting genetic susceptibility. Familial pulmonary fibrosis (FPF) is diagnosed when at least two first- or second-degree blood relatives are affected by ILD 11. FPF accounts for 5-20% of all IPF cases 12, 13. Approximately one-third of individuals with sporadic IPF have a family history of pulmonary fibrosis 14, with nearly a quarter of the genetic risk attributable to rare variants of known FPF-associated genes 13.

**Figure 1:**
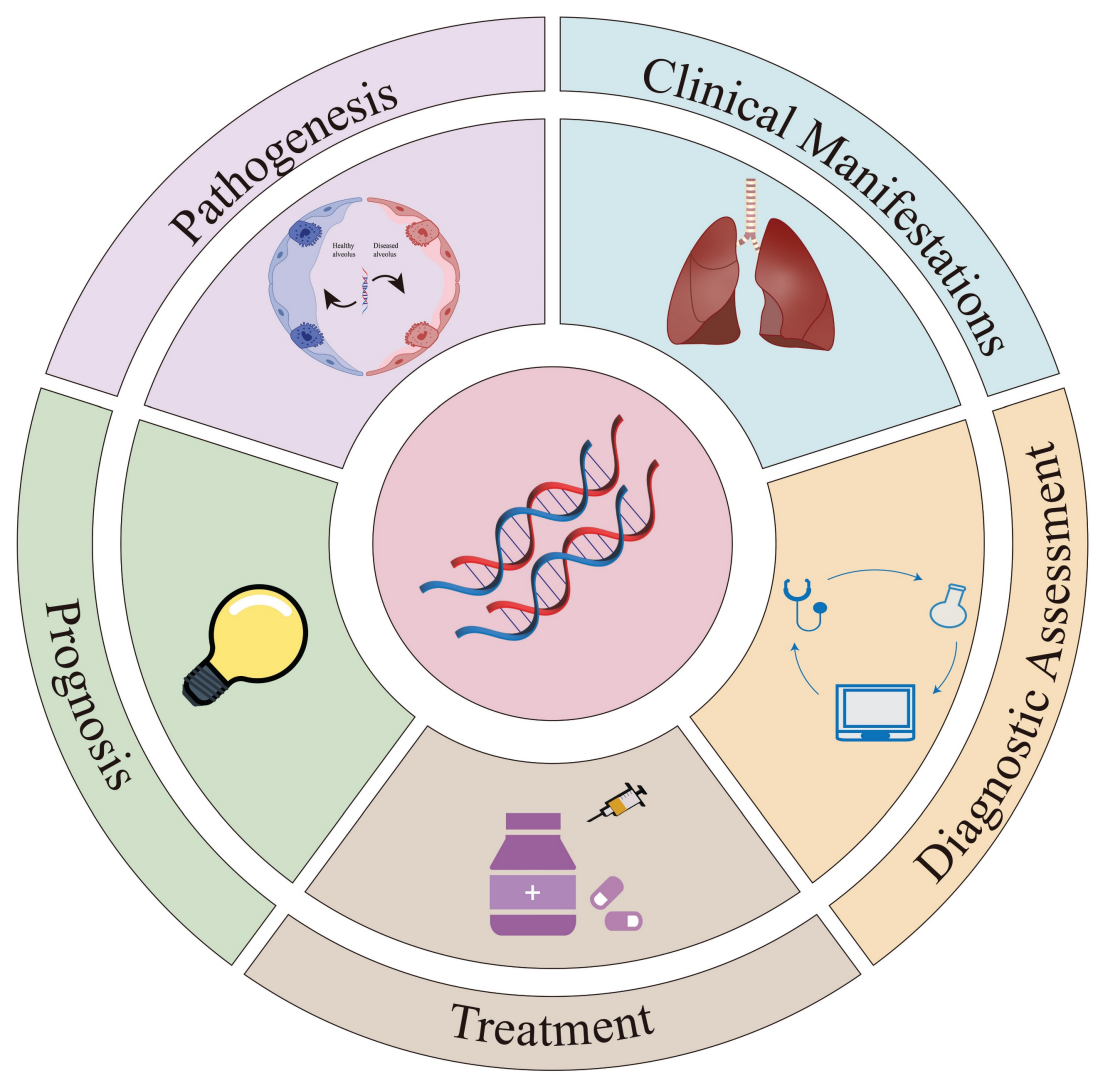
** Graphical summary.** This graphical summary provides an overview of the main topics covered in our review. The “Pathogenesis” section examines several key genetic variations associated with primary profibrotic mechanisms. As shown in Figure 2, genetic factors are closely associated with the progression of IPF. The “Clinical Manifestations” section illustrates the impact of genetic variations on the clinical presentation of IPF patients, particularly regarding lung function. The “Diagnostic Assessment” section examines the importance of genetic factors in IPF diagnosis and predicts their role in future diagnostic strategies. In the “Treatment” section, we analyze the impact of genetic variations on pharmacological and transplantation therapies and evaluate the potential of gene therapy. Finally, the “Prognosis” section proposes innovative methods for developing prognostic models and biomarkers based on genetic factors.

Genetic factors play a role in the development and progression of IPF (Figure 1). While the pathogenesis of IPF remains unclear, it is influenced by a complex interplay of environmental and host factors. Current genetic research has identified key contributors such as mucin 5B (MUC5B) 15, telomerase 5, surfactant proteins 17, cytokines, and related signaling pathways. Moreover, the role of epigenetic signaling pathways in regulating the development and progression of IPF has garnered increased attention. Advances in genetics have further deepened understanding of the pathophysiology of IPF and supported the development of personalized medical strategies. Treatment options for IPF are limited and currently consist of two anti-fibrotic drugs, pirfenidone and nintedanib, which slow disease progression but fail to reverse established fibrosis 10, 18, 19. Lung transplantation may be considered for patients with end-stage disease. Notably, genetic variations, including *MUC5B* promoter polymorphisms and the Desmoplakin (*DSP*) rs2076295 genotype, have been linked to differential therapeutic responses to pirfenidone and nintedanib 20, 21. The genetic perspective improves both the effectiveness of treatments and the quality of life of patients through targeted therapeutic interventions.

In summary, advancements in genetics have provided new perspectives for a deeper understanding of IPF. In this review, we examine the complex role that genetic factors have in the pathogenesis, progression, and prognosis of the disease. While this review emphasizes genetic and epigenetic drivers of IPF, we contextualize these findings with select transcriptomic and proteomic studies that elucidate functional consequences of genetic perturbations. In addition, we present the latest concepts that are useful for researchers to discover new diagnostic and therapeutic pathways.

## 2. Epidemiology

The global incidence of IPF demonstrates marked geographic heterogeneity, with multinational registry studies highlighting distinct epidemiological patterns 25. Notably, adjusted incidence estimates range from 3.5 to 13 per 100,000 individuals in the Asia-Pacific region compared to 0.9-4.9 in Europe and 7.5-9.3 in North America 26. While occupational exposures and environmental factors have been associated with disease risk 27, the strong familial clustering observed in 5-20% of cases provides compelling evidence for genetic predisposition 11.

Emerging genetic epidemiology reveals population-specific risk architectures. For instance, the frequency of the *MUC5B* rs35705950 minor allele differs significantly across populations: 0.007 in East Asian, 0.02 in African/African American, and 0.11 in European populations. While this variant confers stronger association in European populations 28, its attenuated association in Asian cohorts like the South Korean IPF registry 29 suggests modifier loci or environmental interactions may shape ethnic-specific risk profiles. These genetic differences parallel clinical disparities. Multicenter studies have shown that African American patients with pulmonary fibrosis are diagnosed, hospitalized, and die at earlier ages than patients of European or Latino ancestry 30. These differences in clinical outcomes among different ethnic groups require a thorough examination of underlying causes, particularly focusing on the impact of genetic variations on the development and progression of IPF.

## 3. Pathogenesis

### 3.1. Mucin and cell adhesion

**Figure 2:**
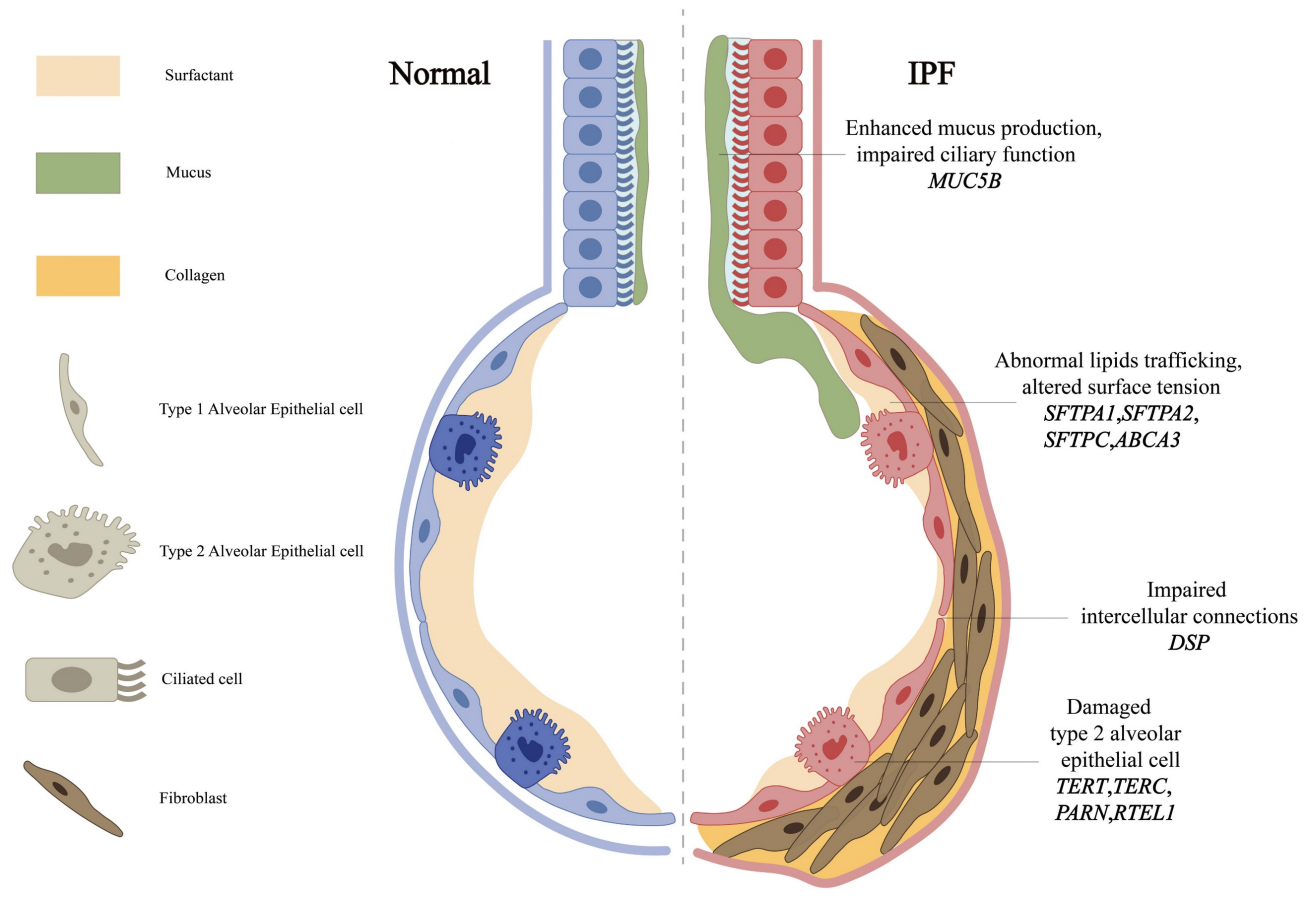
** Some genes related to the main pro-fibrotic mechanisms.** The primary profibrotic mechanisms are associated with both mutations and polymorphisms in genes such as *MUC5B*, *DSP*, telomerase-associated genes (including *TERT, TERC, PARN* and *RTEL1*) and surfactant proteins (including *SFTPA1, SFTPA2, SFTPC* and *ABCA3*). Overexpression of *MUC5B* may impair mucosal defense by reducing the efficiency of ciliary clearance of inhaled particles and microorganisms. Mutations within the* DSP* gene reduce its expression, while DSP is essential for maintaining cell-cell connections and tissue structural integrity. Reduced telomerase activity, due to mutations in *TERT, TERC, PARN* and *RTEL1*, leads to telomere shortening. Surfactant proteins play a critical role in modulating host defense functions, including the production of pro-inflammatory cytokines, cellular chemotaxis, and tissue repair, while also maintaining alveolar stability.

The *MUC5B* gene, encoding a mucin critical for airway defense, is expressed in the mucous glands of the terminal and conducting airways, alveolar type II epithelial cells (AECII), and the epithelial cells of alveolar cysts 31. The T/G variant rs35705950 in its promoter is the strongest genetic risk factor for IPF (Table 1) 32, with epigenetic studies revealing this risk allele is associated with *MUC5B* promoter hypomethylation and transcriptional activation 2. Epigenome-wide studies reinforce DNA methylation's role, such as *MUC5B* promoter hypomethylation, and highlight Solute Carrier Family 6 Member 6 (*SLC6A6*) rs112271207, a taurine transporter with putative epigenetic effects, as a candidate gene 2. While its minor allele frequency varies, functional studies reveal conserved pathogenic mechanisms: excessive production of MUC5B may impair mucosal defense mechanisms and reduce the effectiveness of mucociliary clearance in removing inhaled particles and microorganisms (Figure 2). Elevated levels of MUC5B mucin can disrupt normal surfactant function in the alveoli and distal airways, potentially resulting in alveolar collapse and inflammatory reactions 33. Although these mechanisms are important in the pathogenesis of IPF, they are not exclusive determinants, and the precise role of *MUC5B* in IPF remains unclear. Recent multi-ancestry meta-analyses have also implicated Mucin 1 (*MUC1*), encoding another transmembrane mucin, as a novel susceptibility locus, highlighting mucin dysregulation as a broader mechanism in IPF pathogenesis 34. While Nitrogen Permease Regulator 3-like Protein (*NPRL3*) rs74614704, a regulator of mTORC1 signaling, further links mucin pathways to fibrotic remodeling 3, 35.

DSP, an integral component of desmosomal structures, is crucial for maintaining intercellular connections and tissue integrity. Mechanistically, *DSP* variants, including rs2076295, disrupt alveolar epithelial integrity (Figure 2) 4. Such dysfunction can trigger abnormal extracellular matrix deposition and accelerate the progression of pulmonary fibrosis 4. Notably, the genetic variations in *MUC5B* and *DSP* genes exhibit significant correlations with DNA methylation 36, suggesting that multiple genetic factors may interact synergistically to influence the development and progression of IPF. These findings position mucin and adhesion pathways as interconnected drivers of IPF pathogenesis.

### 3.2. Telomere shortening and dysfunction

Telomeres, protective nucleoprotein complexes at chromosome termini, progressively shorten with cell division. Critical telomere attrition triggers DNA damage response (DDR) pathways. In IPF, sustained DDR activation may compromise the function of AECs, inducing alveolar epithelial cell senescence 37. Mutations in telomerase reverse transcriptase (*TERT*), telomerase RNA component (*TERC*) 6*,* poly(A)-specific ribonuclease (*PARN*), and regulator of telomere length 1 (*RTEL1*) affect telomere length and stability and are associated with the development of pulmonary fibrosis (Table 1) 38. Notably, *TERT* expression in lung fibroblasts correlates with acetylated histone H3K9 binding at its promoter 39, suggesting epigenetic regulation of telomerase activity.

In patients with IPF, a novel mutation in the NHP2 ribonucleoprotein (*NHP2*) gene (p.Y24N) disrupts the nuclear import of the NHP2 protein, thereby reducing the levels of proteins critical for telomere maintenance and telomerase activity 40. Mechanistically, telomere dysfunction impairs AECII progenitor function: dysfunctional telomeres in AECII (Figure 2) disrupt differentiation and regenerative capacity, promoting alveolar collapse and fibrosis 41. Genome-wide association studies (GWAS) now extend telomere-related genetics beyond core telomerase components. For example, the Spindle Apparatus Coiled-coil Protein 1 (*SPDL1*) rs116483731, encoding a mitotic spindle assembly protein, has been identified as a novel risk factor that accelerates telomere attrition and cellular senescence, likely via mitotic errors that exacerbate replicative stress 8. Similarly, Kinetochore Scaffold 1 (*KNL1*) rs12912339 and Stathmin 3 (*STMN3*) rs76537958 variants, implicated in spindle assembly, suggest that mitotic errors contribute to alveolar stem cell dysfunction 3, 32.

While these mutations are associated with IPF, they are not detected in the majority of patients 42. Strikingly, in IPF patients homozygous for the non-risk *MUC5B* rs35705950 allele, rare functional variants in *TERT*, *PARN*, *TERC*, or *RTEL1* are enriched 7, suggesting a potential genetic interaction between *MUC5B* and telomerase-related genes. It highlights the need for stratified genetic testing: *MUC5B*-centric screening may overlook telomerase-driven subtypes requiring distinct management. These findings underscore the interplay between genetic, epigenetic, and mitotic stress mechanisms in IPF pathogenesis.

### 3.3. Surfactants

Pulmonary surfactants, lipid-protein complexes synthesized by AECII, maintain alveolar integrity by reducing surface tension. Surfactant dysfunction directly contributes to IPF pathogenesis through AECII injury and aberrant repair 43 (Figure 2), leading to decreased alveolar stability, alveolar collapse, and the development of fibrosis. Beyond structural roles, surfactant proteins modulate host defense by regulating pro-inflammatory cytokines, chemotaxis factors, and tissue repair processes 43.

Four surfactant-associated genes—surfactant protein A1 (*SFTPA1*), surfactant protein A2 (*SFTPA2*), surfactant protein C (*SFTPC*) and the ATP-binding cassette-type family A member 3 transporter (*ABCA3*)—are implicated in familial and sporadic pulmonary fibrosis (Table 1) 17, 44. Distinct functional roles of *SFTPA1* and *SFTPA2* may arise from altered expression ratios that modulate surfactant activity 9. Autosomal dominant *SFTPC* mutations, like *SFTPC-I73T*, disrupt lamellar body maturation in AECII, causing surfactant accumulation and oxidative stress 16. Recessive *ABCA3* variants, localized to lamellar body membranes, are frequently linked to pediatric interstitial lung disease, underscoring surfactant dysregulation as a pan-age mechanism 17.

### 3.4. Cytokine and signaling pathway dysregulation

The transforming growth factor-β (TGF-β) signaling pathway constitutes a central axis in IPF pathogenesis, with both genetic predisposition and downstream effector mechanisms contributing to fibrotic progression. TGF-β1 induces fibroblast-to-myofibroblast differentiation through canonical Smad2/3 activation 45, 46, with epigenetic mechanisms critically modulating this process. Specifically, TGF-β1 promotes methylation of the Thy-1 promoter and recruits methyl-CpG binding domain protein 2 (MBD2) to activate the TGF-β-Smad pathway, creating a feed-forward loop that sustains fibroblast activation 47. Furthermore, TGF-β1 modulates H3K9me2/3 and H3K4me1/2/3 histone marks at Collagen Type I Alpha 1 (*Col1A1*), Connective Tissue Growth Factor (*CTGF*), and Plasminogen Activator Inhibitor 1** (***PAI-1*) promoters, enhancing the transcriptional activity of fibrosis-associated genes 48. Nuclear accumulation of Smad proteins enables their function as transcriptional regulators of fibrosis-associated genes. Smad complexes also interact with histone deacetylases to remodel chromatin, facilitating extracellular matrix (ECM) protein expression 49. Experimental evidence indicates that Bone Morphogenetic Protein 4 (BMP4) antagonizes TGF-β1-driven effects by activating Smad1/5/9, thereby suppressing Smad2/3 phosphorylation and inhibiting myofibroblast differentiation and ECM synthesis 46. In addition, miR-17-92 and miR-29 inhibit TGF-β-driven fibrosis: miR-17-92 maintains alveolar homeostasis by blocking fibroblast activation 50, while miR-29 suppresses ECM synthesis via Yes-associated protein (YAP) signaling 51.

Canonical Wnt/β-catenin signaling exhibits spatial specificity in IPF, with alveolar epithelial-specific activation driving IL-1β-mediated TGF-β amplification 52. In addition, the non-canonical Wnt signaling pathway, exemplified by WNT5A, initiates cytoskeletal reorganization via the JNK and ROCK signaling pathways. F-actin-generated biomechanical tension facilitates proteolytic activation of latent TGF-β via integrin αv 53. Furthermore, genetic variants in A-kinase anchoring protein 13 (*AKAP13*), a kind of RhoA regulator, exacerbate TGF-β dysregulation and increased IPF susceptibility, underscoring the role of genetic predisposition in fibrotic remodeling 54. Notably, miR-26a downregulation in fibrotic environments exacerbates TGF-β1-induced ECM deposition, whereas its overexpression attenuates fibrosis 55, suggesting miRNA-based modulation of Wnt-TGF-β crosstalk. Emerging evidence positions mTOR signaling as both a TGF-β effector and independent genetic risk modulator. TGF-β-induced mTOR signaling depends on the canonical Smad signaling pathway and is independent of Phosphoinositide 3-kinase (PI3K)/AKT activity 56. Notably, genetic polymorphisms in DEP domain-containing mTOR-interacting protein (DEPTOR), an endogenous mTOR inhibitor, and other mTOR pathway components, like Regulatory Associated Protein of mTOR Complex 1 (RPTOR), have been linked to altered IPF risk, emphasizing the genetic modulation of mTOR-driven fibrosis 57. This multilayered interplay underscores the need for integrated therapeutic approaches to address signaling dysregulation in IPF.

The pathogenesis of IPF involves genetic susceptibility, alveolar epithelial dysfunction, and dysregulated signaling pathways. Mucin/adhesion abnormalities, telomere attrition, and surfactant dysregulation drive injury and senescence. Concurrently, cytokine/signaling imbalances and epigenetic modifications establish fibrotic cascades through genetic-epigenetic crosstalk. These mechanisms underscore the need for stratified therapies targeting molecular subtypes.

## 4. Clinical manifestations and diagnostic assessment

### 4.1. Clinical manifestations

IPF is a chronic and progressive lung disease characterized by a range of clinical symptoms. Initially, patients often experience exertional dyspnea, which may progress to persistent dry cough, weight loss, and other systemic symptoms as the disease advances. On physical examination, findings may include clubbing of the fingers and basal inspiratory crackles or rales, indicating impaired gas exchange and progressive pulmonary fibrosis. High-resolution computed tomography (HRCT) images typically reveal characteristic IPF findings, such as reticular opacities and honeycomb changes. Pulmonary function tests often demonstrate restrictive ventilatory defects and reduced carbon monoxide diffusion capacity. These tests are crucial for diagnosing IPF and monitoring disease progression.

With the advancement of precision medicine, genetic testing of patients with IPF is increasing. Genetic backgrounds can significantly influence the range and severity of clinical manifestations. For example, the* MUC5B* gene encodes key mucins present in the honeycomb cysts of patients with IPF 31. Carriers of the *MUC5B* promoter variant rs35705950 often exhibit more severe coughing symptoms and higher quantitative CT fibrosis scores, which may help quantify disease risk for relatives 58. These variants also correlate with distinct prognoses 59. In addition, further advances in imaging technology may lead to more accurate prognoses for patients. Observational studies show that patients with *SFTPC* or other surfactant-related gene mutations present with atypical radiological patterns, including cystic changes characteristic of interstitial pneumonia 60. These patients are often younger (average age 45 years) and exhibit lower forced vital capacity (FVC) and lung diffusion capacity for carbon monoxide (DLCO) compared to those with familial or sporadic IPF 61. Carriers of *TERT* mutations, even asymptomatic individuals, display significantly reduced DLCO and impaired DLCO response during exercise. HRCT scans in these patients reveal signs of pulmonary fibrosis and increased lung tissue volume fractions. Compared to non-carriers, *TERT* mutation carriers with IPF show a more pronounced reduction in lung diffusion capacity 62.

### 4.2. Diagnostic assessment

The 2018 guidelines 63 jointly published by the American Thoracic Society (ATS), the European Respiratory Society (ERS), the Japanese Respiratory Society (JRS), and the Asociación Latinoamericana de Torax (ALAT), recommend an initial assessment for suspected cases of IPF, with a focus on identifying possible known causes of ILD. After identifying a potential cause of ILD, a comprehensive evaluation is necessary to identify or rule out conditions such as hypersensitivity pneumonitis, connective tissue disease, pneumoconiosis, and iatrogenic problems. If diagnostic uncertainty persists after standard assessments, a multidisciplinary discussion (MDD) should be convened. This MDD should integrate clinical and HRCT findings to confirm or rule out the diagnosis. A definitive IPF diagnosis can be established by correlating appropriate HRCT and histopathological patterns.

Given the overlapping clinical features of chronic respiratory diseases, early differential diagnosis and the identification of relatives at risk for IPF are of significant value in genetic research. A machine learning model that uses gene expression data from peripheral blood mononuclear cells to predict IPF has been developed 64. This 44-gene model can accurately predict IPF in healthy controls and patients with tuberculosis, HIV, and asthma. Moreover, the model also allows subtyping of IPF subtypes. Thus, the model shows promise as a non-invasive diagnostic tool 64. Clinical research has shown that pathogenic variations in the telomerase complex genes are found in approximately 10% of patients with IPF, regardless of family history, suggesting the need for genetic counseling for all patients with IPF 65.

When assessing lung diseases, HRCT provides finer images than traditional CT scans. However, interpreting the morphology and extent of lesions using HRCT remains a relatively subjective process that requires a solid foundation in imaging knowledge, and robust quantitative diagnostic methods are lacking. Additionally, cost and radiation exposure must also be considered. Future genetic research could complement HRCT by establishing quantitative standards for the diagnosis of IPF 58, 66. The *MUC5B* gene is particularly interesting in this context. Polygenic risk scores for IPF and interstitial lung anomalies have been developed using data from a GWAS in IPF, confirming the predictive value of *MUC5B* in identifying individuals at risk for pulmonary fibrosis 67.

## 5. Treatment

### 5.1. Drug therapy

Pirfenidone and nintedanib are anti-fibrotic drugs approved by the Food and Drug Administration (FDA) and the European Medicines Agency (EMA) for the treatment of IPF. Clinical studies have shown that pirfenidone can slow the decline in FVC 18. However, its clinical effectiveness may vary among patients with IPF and different genetic backgrounds. In particular, a subset of patients with IPF who have high expression of genes related to ciliogenic epithelial cells responds more positively to pirfenidone 68. Nintedanib is a small-molecule tyrosine kinase inhibitor that reduces lung fibroblast proliferation, migration, and differentiation by inhibiting multiple growth factor receptors. Nintedanib may slow the rate of FVC decline in patients with IPF and prolong their survival 19. In a cohort analysis, patients carrying the *DSP* rs2076295 G allele experienced greater benefits in overall survival and lung function when treated with nintedanib compared with TT homozygous patients 69. To the best of our knowledge, no studies have specifically examined the association between genetic differences and adverse reactions to these two anti-fibrotic drugs in patients with IPF.

Although clinical trials have confirmed the effectiveness of these drugs in slowing lung function decline 18, 19, they cannot reverse or resolve existing fibrosis. Advances in genetic research have provided new insights into the antifibrotic mechanisms of pirfenidone and nintedanib, and evidence suggests that different gene expression patterns can influence drug efficacy 70. For instance, the Toll-interacting protein (*TOLLIP*) rs3750920 polymorphism has been shown to modify responses to N-acetylcysteine (NAC), with TT genotype carriers deriving therapeutic benefit while CC genotype carriers may experience harm 22. Similarly, the *MUC5B* rs35705950 variant has been linked to differential outcomes in IPF therapies 71. These findings underscore the potential of pharmacogenomics to guide personalized treatment strategies, as exemplified by the ongoing PRECISIONS trial (NCT04300920), which stratifies IPF patients by *TOLLIP* genotypes to optimize NAC therapy 22. Identifying genotypes associated with drug responses could enable the prediction of individual patient responses to these drugs. Furthermore, this may facilitate the development of personalized treatment strategies, including tailored drug selection, dosage adjustment, and combination therapies.

### 5.2. Lung transplantation

The pharmacological treatment of patients with IPF is primarily aimed at slowing the progression of fibrosis and providing palliative care for those in advanced stages. However, despite optimal therapeutic interventions, lung function in patients with IPF may still progressively deteriorate. IPF can reach a terminal stage characterized by severely impaired lung function that is unresponsive to medical treatment. For these patients, lung transplantation (LT) represents the sole therapeutic option capable of significantly prolonging their survival and enhancing quality of life. The 5-year survival rate following lung transplantation approaches 50% 72. Nonetheless, LT faces several obstacles, including the shortage of donor lungs, stringent technical requirements, and the substantial costs associated with the procedure. Additionally, genetic factors have been shown to influence both the success and quality of LT outcomes.

The risk of complications following LT is associated with post-transplant survival and represents a crucial consideration when assessing LT candidacy 73. Clinical observations indicate that lung transplant recipients with IPF (IPF-LTRs) have circulating T cells suggestive of immunodeficiency 74, thereby increasing their risk of hematological complications 75. Moreover, IPF-LTRs are more susceptible to rare telomere-related genetic variants and shorter telomere lengths compared to non-transplant individuals 76. Some researchers have recommended incorporating genetic factors into LT evaluations, such as measuring telomere length prior to transplantation and conducting genetic testing for telomere gene variants. Such tests may help identify IPF transplant recipients who are at higher risk of hematologic complications 75.

The results of a retrospective study indicate that LT is appropriate even in patients with telomerase-related gene mutations, provided there are no myelodysplastic symptoms and a systematic hematological evaluation is performed 77. In addition, genetic factors influence decisions regarding the administration of immunosuppressants after LT. The evidence suggests that standard immunosuppressive therapy should be maintained in young transplant recipients with shorter telomeres, even if immune deficiency is present 74.

## 6. Prognosis

Along with FVC and DLCO, age and gender are important prognostic indicators during the stable phase of IPF. Men generally exhibit worse survival rates than women 78. The GAP staging system integrates age, gender, and lung function parameters (FVC, DLCO) to predict mortality risk, though its sensitivity for short-term (1-year) outcomes remains limited 79. Biomarkers and genetic variations provide complementary prognostic insights and may enhance existing prognostic assessment tools by addressing their limitations.

A retrospective study involving a European cohort of 1751 patients with IPF revealed that the *MUC5B* T allele is a significant independent predictor of patient survival. No significant correlation was observed in patients under 56 years of age, whereas in the older cohort, individuals with the T allele exhibited better survival rates 80. Conversely, in another retrospective case-control study conducted within the Portuguese population, no associations were found between *MUC5B* variations and disease survival rates 81. These inconsistencies likely stem from confounding factors that were not accounted for in the study design or analysis. However, the preponderance of evidence suggests that the *MUC5B* minor T allele is associated with improved patient survival rates 81-83, independent of age, gender, FVC, and DLCO. This paradoxical association (increased disease risk but better survival) may be influenced by index bias, as studies predominantly including prevalent rather than incident IPF cases could disproportionately select for resilient individuals with the *MUC5B* risk allele, thereby inflating survival estimates 84. This underscores the significance of the *MUC5B* genotype in a survival prognosis model for patients with IPF 83. Telomere length has been independently associated with transplant-free survival in patients with IPF, as confirmed by observational cohort studies, further highlighting its role in the prognosis of IPF 85. Ethnic-specific outcomes are evident in the prognosis of IPF patients. Rare *TERT* variants enriched in Latin American IPF patients correlate with aggressive disease trajectories, though mechanistic links require further study 86. Japanese patients show distinct causes of death, like acute exacerbations, and prognoses compared to other ethnic groups 87.

A comprehensive proteomic analysis of multiple IPF patient cohorts has yielded a model based on the different expression levels of osteopontin (OPN), serum protein D (SPD), intercellular adhesion molecule 1 (ICAM1), and matrix metalloproteinase 7 (MMP7), allowing robust differentiation between progressive and stable IPF 88. These circulating serum proteins are significantly associated with clinical outcomes, increased mortality rates, and greater disease severity, highlighting the feasibility of developing serology-based methods to assess IPF progression 88. For instance, baseline serum levels of cathepsin B (CTSB) strongly correlate with the extent of lung function decline at one year. Patients with elevated serum CTSB levels are more likely to exhibit a progressive IPF phenotype, irrespective of GAP stage 89. Given the growing number of potential biomarkers, developing robust methods to evaluate their clinical utility is critical. The innovative progression index offers a quantitative measure of biomarkers' influence on clinical progress 88.

Recent studies have identified polymorphisms in *TGF-β_1_*, proprotein convertase subtilisin/kexin type 6 (*PCSK6*), and protein kinase N2 (*PKN2*) as critical determinants of disease progression and survival outcomes in IPF. The *TGF-β_1_* T869C variant has been implicated in disease severity, with the TT genotype linked to reduced PaO₂ and increased D(A-a)O₂ at diagnosis, suggesting a role in accelerating functional decline 90, 91. Similarly, the *PCSK6* rs35647788 variant has been associated with reduced transplantation-free survival, potentially through dysregulated proteolytic processing of profibrotic mediators 23. The *PKN2* rs115982800 variant, located in the antisense RNA PKN2-AS1, correlates with rapid FVC decline, highlighting its role in cytoskeletal remodeling and fibroblast activation 24. These findings underscore the importance of genetic variants in modulating IPF progression.

Genes associated with endoplasmic reticulum stress 92, macrophage function 93, and mitochondrial dynamics 93 are implicated in the development and progression of IPF. These genes exhibit robust associations with canonical signaling pathways, including the apoptosis signaling pathway and the PI3K/AKT pathway, which collectively modulate the pulmonary immune microenvironment. Prognostic signature genes derived from the synergistic expression of m5C-regulated genes and immune-associated genes are likely to exert significant influence over immune and inflammatory responses, enabling precise prediction of survival outcomes in IPF patients 94. Continued advancements in genetic research are anticipated to unveil novel genetic determinants, thereby facilitating the development of refined prognostic models and elucidating the underlying mechanisms of IPF pathogenesis.

## 7. Genetic-Based Diagnosis and Treatment Models

Genetic testing serves principally as an auxiliary tool within the classical diagnostic and therapeutic strategies for IPF. Although genetic testing exhibits limited independent utility for IPF diagnosis, treatment, or prognosis, its integration as a central analytical framework in clinical practice remains feasible. This strategy is particularly advantageous for patient subgroups at elevated risk of disease progression and adverse outcomes, as it enables the customization of therapeutic strategies according to individual genetic profiles.

**Figure 3:**
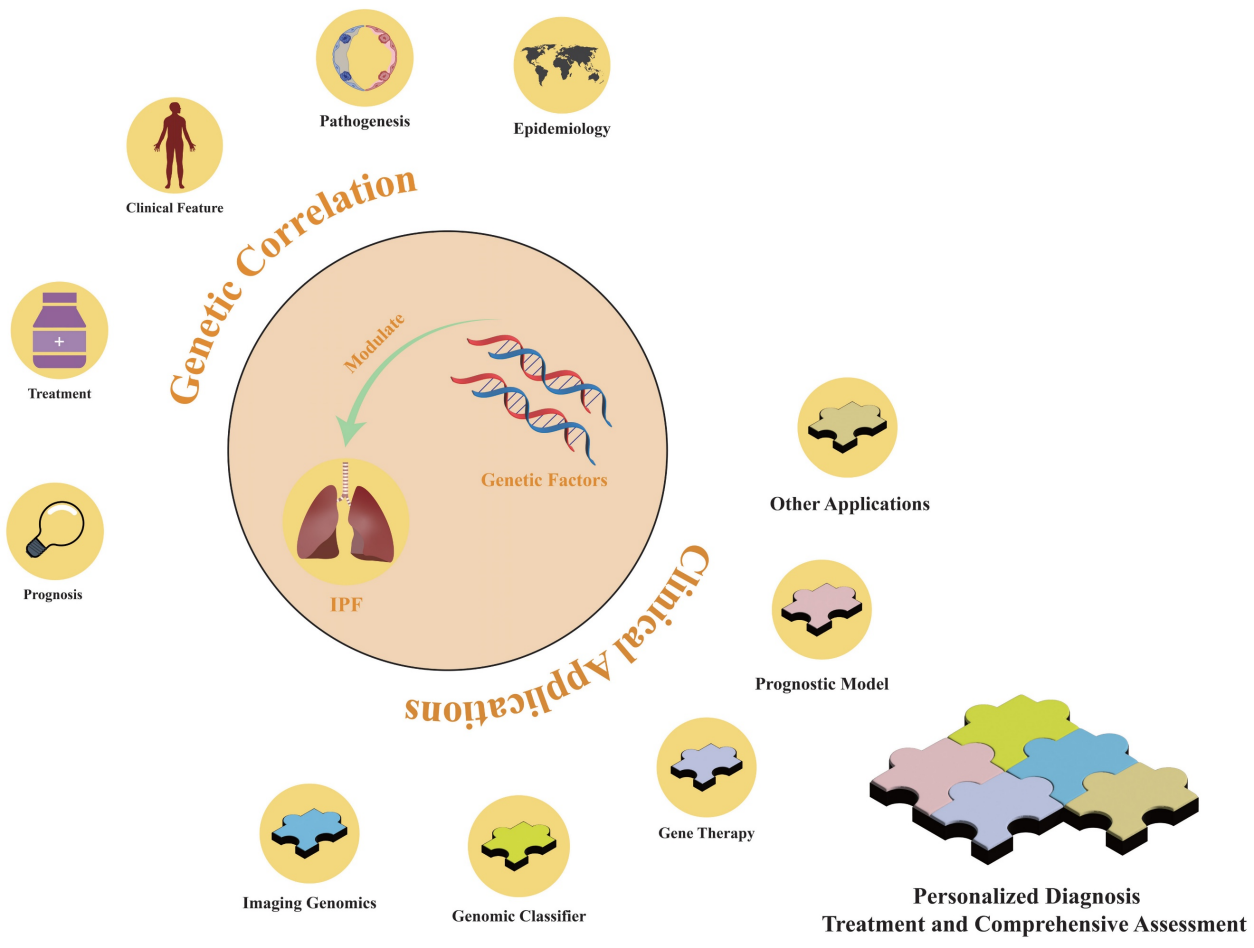
** The role of genetic factors in idiopathic pulmonary fibrosis.** Genetic factors are implicated in the epidemiology, pathogenesis, clinical manifestations, diagnostic approaches, therapeutic modalities, and prognostic outcomes of IPF. By leveraging genetic factors, personalized diagnostic methods and comprehensive assessment models for treatment and prognostic benefits can be developed.

Genetic studies have delineated distinct patient subgroups for IPF diagnosis that can be used to identify high-risk populations. The 2022 ATS/ERS/JRS/ALAT guidelines emphasize the significance of genetic factors in stratifying the clinical severity of IPF and discuss their clinical applications. Despite guideline recommendations against using genomic classifiers as a standard diagnostic tool for usual interstitial pneumonia due to a lack of consensus 10, genetic factors remain essential in the personalized treatment strategies of precision medicine. As advancements in genetic etiology and cost-effective genetic testing continue, identifying patients who would benefit from genetic testing will grow increasingly critical. This will aid in developing intervention strategies to slow disease progression and selecting the most appropriate screening and management protocols (Figure 3). The Envision Genomic Classifier for IPF diagnosis, a clinically validated tool derived from whole transcriptome mRNA sequencing in transbronchial biopsy samples, integrates clinical factors and HRCT imaging, demonstrating high diagnostic sensitivity 95. Moreover, detecting the differential expression of the pirfenidone response gene across IPF subgroups is important, and sophisticated machine learning techniques can facilitate the development of classifiers that reflect cell-type characteristics and gene expression patterns 68.

Genetic variations in patients with IPF influence their responsiveness to pharmacotherapies, necessitating the integration of genetic insights into drug development (Figure 3). Modern genomics offers precise molecular targets for disease diagnosis and therapeutic intervention. Integrating gene expression profiles and pathological characteristics of patients with IPF into computational methods can expedite drug development 96. Artificial intelligence platforms that identify drug targets optimize the drug development process by streamlining target discovery 97. For instance, computational simulations have demonstrated that a plant-derived microRNA, osa-miR172d-5p, downregulates the expression of TAK1-binding protein 1 and fibrosis-related genes in TGF-β-stimulated pulmonary fibroblasts 98.

In murine studies, gene therapy has demonstrated the potential to arrest the progression of pulmonary fibrosis. Utilizing an adeno-associated virus serotype 9 (AAV9)-Tert vector for gene therapy, reactivation of telomerase in the lung can delay disease progression in murine models of pulmonary fibrosis 99. Prophylactic intratracheal administration of AAV9-Tspyl2 delays the onset of bleomycin-induced pulmonary fibrosis in mice by inhibiting the TGF-β/Smad3 signaling pathway 100. *BIX01294*, an exceptionally selective G9a histone methyltransferase inhibitor, reduces TGF-β-induced H3K9 methylation and matrix stiffness via upregulation of the *PPARGC1A* gene 101, thereby diminishing collagen deposition in the lungs of mice following bleomycin injury. These results suggest that therapeutic interventions targeting epigenetic repression mechanisms hold promise. Studies on the relaxin/RXFP1 axis 102 and microRNA-144-3p 103 have revealed their promising anti-fibrotic properties in patients with IPF, offering new avenues for IPF therapeutics.

The identification of genetic factors enables the stratification of IPF cases, the correlation of genetic and phenotypic profiles, and prediction of treatment effectiveness and patient outcomes (Figure 3). The molecular signatures of IPF-associated fibroblastic subtypes and their prognostic implications have been characterized through machine learning and single-cell analyses 104. New risk assessment models can be developed using bioinformatics and machine learning algorithms to facilitate the stratification of patient subgroups and refine personalized therapeutic strategies.

## 8. Discussion

Genetic factors significantly contribute to the epidemiological variance observed in IPF and are significant to its pathogenesis. The incidence and clinical outcomes of IPF exhibit variability across ethnicities and regions, primarily due to genetic predispositions. For instance, in Asian and North American populations, the minor allele frequency of the T allele in the *MUC5B* gene rs35705950 is significantly correlated with the IPF incidence. However, the lower incidence of IPF in the European population, despite a higher frequency of the minor T allele, suggests that additional factors affect disease development. In addition to genetic factors, environmental determinants, including occupational exposure and gender differences, have been implicated in the occurrence and progression of IPF (Figure 3). A broad consensus exists that environmental and host factors exert cumulative effects on IPF risk 105, and family history research offers an opportunity to elucidate the genetic contributions to IPF.

Family studies have revealed the so-called anticipation phenomenon, characterized by an earlier onset of pulmonary fibrosis symptoms in successive generations of families with *TERT* mutations. This phenomenon correlates with shorter telomeres in the offspring of families with telomerase mutations, which have been validated as a risk factor for IPF and are associated with adverse clinical outcomes 106. Although anticipation has not been observed for other genetic factors implicated in the etiology and progression of IPF (such as *MUC5B*, *DSP*, and surfactant proteins), evidence suggests potential interactions among these factors 7, 33. Epigenetic mechanisms may act both as independent contributors to IPF 36 and as mediators in its pathogenesis 36, 39. A bioinformatics-driven network of genetic interactions may systematically elucidate the role of genetic factors in IPF pathogenesis.

Genetic variations are important factors in the lung function and radiological features of patients with IPF, influencing the spectrum and severity of clinical phenotypes. Most patients with IPF experience a gradual decline in clinical, functional, and radiographic status, but some undergo acute respiratory exacerbations (AE-IPF). Research indicates that shortened telomere length is associated with an increased risk of AE-IPF or mortality in these patients 107, and that expression of the *S100A8/A12* genes is linked to the etiology of AE-IPF 108. Genetic factors provide important insights into the stratification of clinical severity in patients with IPF, as acknowledged in the ATS/ERS/JRS/ALAT clinical guidelines, which recognize the clinical application of genetic classifiers 10. Physicians are encouraged to thoroughly investigate the optimal application of genetic factors to enhance disease diagnosis and treatment, in accordance with current clinical guidelines.

Genetic imaging, a multidisciplinary field integrating genetics and neuroimaging, assesses changes in brain morphology and function to determine the impact of genetic variations on individual behaviors and diseases. Imaging techniques have identified links between genetic variations and brain structures, for example, a correlation has been found between the SNP rs42352 in the Semaphorin 5A (*SEMA5A*) gene and bilateral hippocampal volume 109. Incorporating imaging data to examine associations between genetic variations and disease enhances diagnostic confidence and demonstrates the viability of a new diagnostic paradigm that leverages genetic factors to interpret imaging data and clinical phenotypes. This approach is particularly relevant in the IPF field, which relies heavily on diagnostic imaging tools (Figure 3). Studying imaging variants caused by genetic factors can highlight the role of imaging data in tracking disease progression. In addition, genetic factors can serve as quantifiable benchmarks and provide complementary assessment support in challenging imaging diagnoses.

In the realm of pharmacotherapy for IPF, FDA-approved treatments such as pirfenidone and nintedanib lack evidence of reversing pulmonary fibrosis, underscoring significant market opportunities for the development of novel IPF therapies. Understanding the genetic factors involved in IPF pathogenesis provides a theoretical basis for leveraging gene therapy to treat the disease. For example, Luxturna, the groundbreaking in vivo gene therapy for hereditary retinal dystrophy, transfers the *RPE65* gene to retinal cells using an AAV vector, and its successful approval confirmed the feasibility of gene therapy 110. While experimental data from clinical trials are limited, AAV-based in vivo gene therapy has demonstrated the potential to halt the progression of pulmonary fibrosis in murine models, representing a promising avenue for IPF treatment.

Prognostic assessments significantly influence physicians' clinical decision-making. In the context of IPF, a disease characterized by complex genetic mechanisms and diverse clinical manifestations, machine learning and bioinformatics may be leveraged to develop personalized prognostic models to overcome the limitations of the GAP tier system in short-term risk prediction. The identification of additional biomarkers that can predict disease progression would be clinically useful, enabling timely adjustments to treatment protocols in response to individual patient progression. Current biomarkers exhibit variable reliability for predicting IPF progression, and identifying robust markers remains a challenge. Computer-assisted techniques that assign weights to each marker in disease progression could streamline the development of future clinical guidelines and facilitate the integration of new biomarkers.

### 8.1. Limitations

This review has several limitations. First, it is not a systematic review and has not undergone a formal quality assessment. Second, the articles included are limited to English-language publications. Third, we focused on describing the role of certain genetic factors in IPF, although some pathogenic mechanisms have not been studied in detail. Fourth, relevant articles may have been overlooked.

## 9. Conclusions

Genetic factors are of utmost importance in the etiology and progression of IPF. They significantly influence the epidemiological profile of IPF and its pathogenesis, clinical presentation, and prognostic outcomes. Current genetic research is poised to refine existing diagnostic frameworks and provide essential quantitative benchmarks for the development of personalized therapeutic strategies. By recognizing the importance of genetic factors and refining diagnostic and therapeutic methods tailored to these influences, it will be possible to better categorize therapeutic interventions and improve the accuracy of prognostic assessments. This approach aligns with the emerging paradigm of precision medicine.

## Figures and Tables

**Table 1:** Genetic variants associated with idiopathic pulmonary fibrosis

Gene	Variant	Chr	Impact on IPF	Reference
MUC5B	rs35705950	11	Strongest genetic risk factor; promoter hypomethylation increases mucin production, impairing mucosal defense. Associated with higher CT fibrosis scores	1
SLC6A6	rs112271207	3	Its role as a taurine transporter suggests epigenetic regulation of IPF pathogenesis	2
NPRL3	rs74614704	16	Regulates mTORC1 signaling, linking mucin pathways (e.g., MUC5B) to fibrotic remodeling. May modulate cellular energy metabolism and fibrotic signaling cascades	3
DSP	rs2076295	6	Disrupts alveolar epithelial integrity, accelerating fibrosis. Correlates with differential responses to nintedanib therapy	4
TERT	rs4449583	5	Telomere shortening induces alveolar epithelial senescence. Linked to early-onset IPF, hematologic complications post-transplant, and reduced survival	5
TERC	rs2293607	3		6
PARN	/	/		7
RTEL1	rs41308092	20		5
SPDL1	rs116483731	5	Risk factor accelerating telomere attrition through mitotic errors	8
KNL1	rs12912339	15	Mitotic spindle assembly protein variant implicated in mitotic errors and replicative stress. Contributes to alveolar stem cell dysfunction and cellular senescence	3
STMN3	rs112087793	20	Variant associated with cytoskeletal reorganization via spindle assembly defects. Promotes mitotic stress, impairing alveolar epithelial repair and regeneration	3
SFTPA1	rs1215316727	/	Mutations cause surfactant dysfunction, leading to alveolar collapse and fibrosis. Associated with atypical radiological patterns and rapid lung function decline	9
SFTPA2	rs371035540	/		9
SFTPC	/	/		16
ABCA3	/	/		17
AKAP13	rs62025270	15	RhoA regulator exacerbates TGF-β dysregulation, increasing fibrotic remodeling	7
TOLLIP	rs3750920	11	Modifies therapeutic response to N-acetylcysteine; TT genotype benefits while CC genotype may worsen outcomes	22
PCSK6	rs35647788	15	Associated with reduced transplant-free survival via dysregulated proteolytic processing of profibrotic mediators	23
PKN2	rs115982800	1	Correlates with rapid FVC decline; regulates cytoskeletal remodeling and fibroblast activation	24

Chr = chromosome.

## Abbreviations

AAV: Adeno-associated virus
AAV9: Adeno-associated virus serotype 9
ABCA3: ATP-binding cassette-type family A member 3 transporter
AEC: Alveolar epithelial cell
AECII: Alveolar type II epithelial cells
AE-IPF: Acute exacerbation of IPF
AKAP13: A-kinase anchoring protein 13
ALAT: Asociación Latinoamericana de Torax
ATS: American Thoracic Society
BMP4: Bone morphogenetic protein 4
Col1A1: Collagen type I alpha 1
CTGF: Connective tissue growth factor
CTSB: Cathepsin B
DDR: DNA damage response
DEPTOR: DEP domain-containing mTOR-interacting protein
DLCO: Diffusion capacity for carbon monoxide
DSP: Desmoplakin
ECM: Extracellular matrix
EMA: European Medicines Agency
ERS: European Respiratory Society
FDA: Food and Drug Administration
FPF: Familial pulmonary fibrosis
FVC: Forced vital capacity
GAP: Gender-age-physiology (staging system)
GWAS: Genome-wide association study
HRCT: High-Resolution Computed Tomography
ICAM1: Intercellular adhesion molecule 1
ILD: Interstitial lung disease
IPF: Idiopathic pulmonary fibrosis
IPF-LTRs: Lung transplant recipients with IPF
JRS: Japanese Respiratory Society
KNL1: Kinetochore scaffold 1
LT: Lung transplantation
MBD2: Methyl-CpG binding domain protein 2
MDD: Multidisciplinary discussion
MMP7: Matrix metalloproteinase 7
mTOR: Mechanistic target of rapamycin
MUC1: Mucin 1
MUC5B: Mucin 5B
NAC: N-acetylcysteine
NHP2: NHP2 ribonucleoprotein
NPRL3: Nitrogen permease regulator 3-like protein
OPN: Osteopontin
PAI-1: Plasminogen activator inhibitor 1
PARN: Poly(A)-specific ribonuclease
PBMC: Peripheral blood mononuclear cells
PCSK6: Proprotein convertase subtilisin/kexin type 6
PI3K: Phosphatidylinositol 3-kinase
PI3K: Phosphoinositide 3-Kinase
PKN2: Protein kinase N2
RPTOR: Regulatory associated protein of mTOR complex 1
RTEL1: Regulator of telomere length 1
SEMA5A: Semaphorin 5A
SFTPA1: Surfactant protein A1
SFTPA2: Surfactant protein A2
SFTPC: Surfactant protein C
SLC6A6: Solute carrier family 6 member 6
SPD: Serum protein D
SPDL1: Spindle apparatus coiled-coil protein 1
STMN3: Stathmin 3
TERC: Telomerase RNA component
TERT: Telomerase reverse transcriptase
TGF-β: Transforming growth factor-β
TOLLIP: Toll-interacting protein
YAP: Yes-associated protein

## References

[1] Seibold MA, Wise AL, Speer MC, Steele MP, Brown KK, Loyd JE (2011). A common MUC5B promoter polymorphism and pulmonary fibrosis. N Engl J Med.

[2] Chin D, Hernandez-Beeftink T, Donoghue L, Guillen-Uio B, Leavy OC, Adegunsoye A (2025). Genome-wide association study of Idiopathic Pulmonary Fibrosis susceptibility using clinically-curated European-ancestry datasets. medRxiv.

[3] Allen RJ, Stockwell A, Oldham JM, Guillen-Guio B, Schwartz DA, Maher TM (2022). Genome-wide association study across five cohorts identifies five novel loci associated with idiopathic pulmonary fibrosis. Thorax.

[4] Hao Y, Bates S, Mou H, Yun JH, Pham B, Liu J (2020). Genome-Wide Association Study: Functional Variant rs2076295 Regulates Desmoplakin Expression in Airway Epithelial Cells. American Journal of Respiratory and Critical Care Medicine.

[5] Peljto AL, Blumhagen RZ, Walts AD, Cardwell J, Powers J, Corte TJ (2023). Idiopathic Pulmonary Fibrosis Is Associated with Common Genetic Variants and Limited Rare Variants. American journal of respiratory and critical care medicine.

[6] Moore C, Blumhagen RZ, Yang IV, Walts A, Powers J, Walker T (2019). Resequencing Study Confirms That Host Defense and Cell Senescence Gene Variants Contribute to the Risk of Idiopathic Pulmonary Fibrosis. American Journal of Respiratory and Critical Care Medicine.

[7] Dressen A, Abbas AR, Cabanski C, Reeder J, Ramalingam TR, Neighbors M (2018). Analysis of protein-altering variants in telomerase genes and their association with MUC5B common variant status in patients with idiopathic pulmonary fibrosis: a candidate gene sequencing study. The Lancet Respiratory Medicine.

[8] Dhindsa RS, Mattsson J, Nag A, Wang Q, Wain LV, Allen R (2021). Identification of a missense variant in SPDL1 associated with idiopathic pulmonary fibrosis. Commun Biol.

[9] Floros J, Thorenoor N, Tsotakos N, Phelps DS (2021). Human Surfactant Protein SP-A1 and SP-A2 Variants Differentially Affect the Alveolar Microenvironment, Surfactant Structure, Regulation and Function of the Alveolar Macrophage, and Animal and Human Survival Under Various Conditions. Frontiers in Immunology.

[10] Raghu G, Remy-Jardin M, Richeldi L, Thomson CC, Inoue Y, Johkoh T (2022). Idiopathic Pulmonary Fibrosis (an Update) and Progressive Pulmonary Fibrosis in Adults: An Official ATS/ERS/JRS/ALAT Clinical Practice Guideline. Am J Respir Crit Care Med.

[11] Borie R, Kannengiesser C, Antoniou K, Bonella F, Crestani B, Fabre A (2023). European Respiratory Society statement on familial pulmonary fibrosis. Eur Respir J.

[12] Raghu G, Collard HR, Egan JJ, Martinez FJ, Behr J, Brown KK (2011). An official ATS/ERS/JRS/ALAT statement: idiopathic pulmonary fibrosis: evidence-based guidelines for diagnosis and management. American Journal of Respiratory and Critical Care Medicine.

[13] Liu Q, Zhou Y, Cogan JD, Mitchell DB, Sheng Q, Zhao S (2023). The Genetic Landscape of Familial Pulmonary Fibrosis. American Journal of Respiratory and Critical Care Medicine.

[14] Moss BJ, Ryter SW, Rosas IO (2022). Pathogenic Mechanisms Underlying Idiopathic Pulmonary Fibrosis. Annual Review of Pathology: Mechanisms of Disease.

[15] Evans CM, Fingerlin TE, Schwarz MI, Lynch D, Kurche J, Warg L (2016). Idiopathic Pulmonary Fibrosis: A Genetic Disease That Involves Mucociliary Dysfunction of the Peripheral Airways. Physiol Rev.

[16] Dickens JA, Rutherford EN, Abreu S, Chambers JE, Ellis MO, van Schadewijk A (2022). Novel insights into surfactant protein C trafficking revealed through the study of a pathogenic mutant. The European respiratory journal.

[17] Sutton RM, Bittar HT, Sullivan DI, Silva AG, Bahudhanapati H, Parikh AH (2022). Rare surfactant-related variants in familial and sporadic pulmonary fibrosis. Human Mutation.

[18] Noble PW, Albera C, Bradford WZ, Costabel U, Glassberg MK, Kardatzke D (2011). Pirfenidone in patients with idiopathic pulmonary fibrosis (CAPACITY): two randomised trials. Lancet (London, England).

[19] Richeldi L, du Bois RM, Raghu G, Azuma A, Brown KK, Costabel U (2014). Efficacy and safety of nintedanib in idiopathic pulmonary fibrosis. The New England Journal of Medicine.

[20] Biondini D, Cocconcelli E, Bernardinello N, Lorenzoni G, Rigobello C, Lococo S (2021). Prognostic role of MUC5B rs35705950 genotype in patients with idiopathic pulmonary fibrosis (IPF) on antifibrotic treatment. Respir Res.

[21] Provenzani A, Leonardi Vinci D, Alaimo M, Di Maria S, Tuzzolino F, Floridia G (2025). Real-world insights into safety, tolerability, and predictive factors of adverse drug reactions in treating idiopathic pulmonary fibrosis with pirfenidone and nintedanib. Ther Adv Drug Saf.

[22] Oldham JM, Ma SF, Martinez FJ, Anstrom KJ, Raghu G, Schwartz DA (2015). TOLLIP, MUC5B, and the Response to N-Acetylcysteine among Individuals with Idiopathic Pulmonary Fibrosis. Am J Respir Crit Care Med.

[23] Oldham JM, Allen RJ, Lorenzo-Salazar JM, Molyneaux PL, Ma SF, Joseph C (2023). PCSK6 and Survival in Idiopathic Pulmonary Fibrosis. Am J Respir Crit Care Med.

[24] Allen RJ, Oldham JM, Jenkins DA, Leavy OC, Guillen-Guio B, Melbourne CA (2023). Longitudinal lung function and gas transfer in individuals with idiopathic pulmonary fibrosis: a genome-wide association study. Lancet Respir Med.

[25] Hutchinson J, Fogarty A, Hubbard R, McKeever T (2015). Global incidence and mortality of idiopathic pulmonary fibrosis: a systematic review. The European Respiratory Journal.

[26] Maher TM, Bendstrup E, Dron L, Langley J, Smith G, Khalid JM (2021). Global incidence and prevalence of idiopathic pulmonary fibrosis. Respiratory Research.

[27] Pauchet A (2022). Idiopathic Pulmonary Fibrosis: What do we Know about the Role of Occupational and Environmental Determinants? A Systematic Literature Review and Meta-Analysis. Journal of toxicology and environmental health Part B, Critical reviews.

[28] Wu X, Li W, Luo Z, Chen Y (2021). The minor T allele of the MUC5B promoter rs35705950 associated with susceptibility to idiopathic pulmonary fibrosis: a meta-analysis. Scientific reports.

[29] Peljto AL, Selman M, Kim DS, Murphy E, Tucker L, Pardo A (2015). The MUC5B promoter polymorphism is associated with idiopathic pulmonary fibrosis in a Mexican cohort but is rare among Asian ancestries. Chest.

[30] Adegunsoye A, Freiheit E, White EN, Kaul B, Newton CA, Oldham JM (2023). Evaluation of Pulmonary Fibrosis Outcomes by Race and Ethnicity in US Adults. JAMA network open.

[31] Kurche JS, Cool CD, Blumhagen RZ, Dobrinskikh E, Heinz D, Herrera JA (2024). MUC5B Idiopathic Pulmonary Fibrosis Risk Variant Promotes a Mucosecretory Phenotype and Loss of Small Airway Secretory Cells. Am J Respir Crit Care Med.

[32] Partanen JJ, Häppölä P, Zhou W, Lehisto AA, Ainola M, Sutinen E (2022). Leveraging global multi-ancestry meta-analysis in the study of idiopathic pulmonary fibrosis genetics. Cell Genom.

[33] Asakura T, Okuda K, Chen G, Dang H, Kato T, Mikami Y (2024). Proximal and Distal Bronchioles Contribute to the Pathogenesis of Non-Cystic Fibrosis Bronchiectasis. American Journal of Respiratory and Critical Care Medicine.

[34] Milara J, Ballester B, Montero P, Escriva J, Artigues E, Alós M (2020). MUC1 intracellular bioactivation mediates lung fibrosis. Thorax.

[35] Donoghue LJ, Stockwell AD, Neighbors M, Sheng RX, Prabhakaran R, Wolters PJ (2023). Identification of a Genetic Susceptibility Locus for Idiopathic Pulmonary Fibrosis in the 16p Subtelomere Using Whole-Genome Sequencing. Am J Respir Crit Care Med.

[36] Borie R, Cardwell J, Konigsberg IR, Moore CM, Zhang W, Sasse SK (2022). Colocalization of Gene Expression and DNA Methylation with Genetic Risk Variants Supports Functional Roles of MUC5B and DSP in Idiopathic Pulmonary Fibrosis. American journal of respiratory and critical care medicine.

[37] McDonough JE, Martens DS, Tanabe N, Ahangari F, Verleden SE, Maes K (2018). A role for telomere length and chromosomal damage in idiopathic pulmonary fibrosis. Respiratory research.

[38] Fingerlin TE, Murphy E, Zhang W, Peljto AL, Brown KK, Steele MP (2013). Genome-wide association study identifies multiple susceptibility loci for pulmonary fibrosis. Nat Genet.

[39] Liu T, Ullenbruch M, Young Choi Y, Yu H, Ding L, Xaubet A (2013). Telomerase and telomere length in pulmonary fibrosis. American journal of respiratory cell and molecular biology.

[40] Liu L, Sheng Y, Wang C-Y, Liu X, Guo T, Peng H (2023). A novel mutation (p.Y24N) in NHP2 leads to idiopathic pulmonary fibrosis and lung carcinoma chronic obstructive lung disease by disrupting the expression and nucleocytoplasmic localization of NHP2. Biochimica et biophysica acta Molecular basis of disease.

[41] Jackson S-R, Lee J, Reddy R, Williams GN, Kikuchi A, Freiberg Y (2011). Partial pneumonectomy of telomerase null mice carrying shortened telomeres initiates cell growth arrest resulting in a limited compensatory growth response. American Journal of Physiology Lung Cellular and Molecular Physiology.

[42] Wang H, Xu H, Lyu W, Xu Q, Fan S, Chen H (2022). KLF4 regulates TERT expression in alveolar epithelial cells in pulmonary fibrosis. Cell death & disease.

[43] Frerking I, Günther A, Seeger W, Pison U (2001). Pulmonary surfactant: functions, abnormalities and therapeutic options. Intensive Care Medicine.

[44] Legendre M, Butt A, Borie R, Debray MP, Bouvry D, Filhol-Blin E (2020). Functional assessment and phenotypic heterogeneity of SFTPA1 and SFTPA2 mutations in interstitial lung diseases and lung cancer. Eur Respir J.

[45] Hata A, Chen Y-G (2016). TGF-β Signaling from Receptors to Smads. Cold Spring Harbor Perspectives in Biology.

[46] Guan R, Yuan L, Li J, Wang J, Li Z, Cai Z (2022). Bone morphogenetic protein 4 inhibits pulmonary fibrosis by modulating cellular senescence and mitophagy in lung fibroblasts. The European respiratory journal.

[47] Wang Y, Zhang L, Huang T, Wu G-R, Zhou Q, Wang F-X (2022). The methyl-CpG-binding domain 2 facilitates pulmonary fibrosis by orchestrating fibroblast to myofibroblast differentiation. The European Respiratory Journal.

[48] Sun G, Reddy MA, Yuan H, Lanting L, Kato M, Natarajan R (2010). Epigenetic histone methylation modulates fibrotic gene expression. Journal of the American Society of Nephrology: JASN.

[49] Ohkouchi S, Kanehira M, Saigusa D, Ono M, Tazawa R, Terunuma H (2022). Metabolic and Epigenetic Regulation of SMAD7 by STC1 Ameliorates Lung Fibrosis. American journal of respiratory cell and molecular biology.

[50] Dakhlallah D, Batte K, Wang Y, Cantemir-Stone CZ, Yan P, Nuovo G (2013). Epigenetic regulation of miR-17~92 contributes to the pathogenesis of pulmonary fibrosis. American journal of respiratory and critical care medicine.

[51] Herrera J, Beisang DJ, Peterson M, Forster C, Gilbertsen A, Benyumov A (2018). Dicer1 Deficiency in the Idiopathic Pulmonary Fibrosis Fibroblastic Focus Promotes Fibrosis by Suppressing MicroRNA Biogenesis. American journal of respiratory and critical care medicine.

[52] Königshoff M, Balsara N, Pfaff E-M, Kramer M, Chrobak I, Seeger W (2008). Functional Wnt signaling is increased in idiopathic pulmonary fibrosis. PLoS One.

[53] Trinh-Minh T, Chen C-W, Tran Manh C, Li Y-N, Zhu H, Zhou X (2024). Noncanonical WNT5A controls the activation of latent TGF-β to drive fibroblast activation and tissue fibrosis. The Journal of clinical investigation.

[54] Allen RJ, Porte J, Braybrooke R, Flores C, Fingerlin TE, Oldham JM (2017). Genetic variants associated with susceptibility to idiopathic pulmonary fibrosis in people of European ancestry: a genome-wide association study. Lancet Respir Med.

[55] Liang H, Xu C, Pan Z, Zhang Y, Xu Z, Chen Y (2014). The antifibrotic effects and mechanisms of microRNA-26a action in idiopathic pulmonary fibrosis. Molecular therapy: the journal of the American Society of Gene Therapy.

[56] Woodcock HV, Eley JD, Nanthakumar C, Maher TM, Mercer PF, Chambers RC (2016). S51 MTOR regulates TGF-β induced pro-fibrotic gene expression in primary human lung fibroblasts. Thorax.

[57] Allen RJ, Guillen-Guio B, Oldham JM, Ma SF, Dressen A, Paynton ML (2020). Genome-Wide Association Study of Susceptibility to Idiopathic Pulmonary Fibrosis. Am J Respir Crit Care Med.

[58] Mathai SK, Humphries S, Kropski JA, Blackwell TS, Powers J, Walts AD (2019). MUC5B variant is associated with visually and quantitatively detected preclinical pulmonary fibrosis. Thorax.

[59] Cocconcelli E, Bernardinello N, Giraudo C, Castelli G, Greco C, Polverosi R (2022). Radiological Assessment in Idiopathic Pulmonary Fibrosis (IPF) Patients According to MUC5B Polymorphism. International journal of molecular sciences.

[60] van Moorsel CHM, van Oosterhout MFM, Barlo NP, de Jong PA, van der Vis JJ, Ruven HJT (2010). Surfactant protein C mutations are the basis of a significant portion of adult familial pulmonary fibrosis in a dutch cohort. American journal of respiratory and critical care medicine.

[61] Klay D, Grutters JC, van der Vis JJ, Platenburg MGJP, Kelder JC, Tromp E (2023). Progressive Disease With Low Survival in Adult Patients With Pulmonary Fibrosis Carrying Surfactant-Related Gene Mutations: An Observational Study. Chest.

[62] Diaz de Leon A, Cronkhite JT, Yilmaz C, Brewington C, Wang R, Xing C (2011). Subclinical lung disease, macrocytosis, and premature graying in kindreds with telomerase (TERT) mutations. Chest.

[63] Raghu G, Remy-Jardin M, Myers JL, Richeldi L, Ryerson CJ, Lederer DJ (2018). Diagnosis of Idiopathic Pulmonary Fibrosis. An Official ATS/ERS/JRS/ALAT Clinical Practice Guideline. American Journal of Respiratory and Critical Care Medicine.

[64] Shapanis A, Jones MG, Schofield J, Skipp P (2023). Topological data analysis identifies molecular phenotypes of idiopathic pulmonary fibrosis. Thorax.

[65] Šelb J, Osolnik K, Kern I, Korošec P, Rijavec M (2022). Utility of Telomerase Gene Mutation Testing in Patients with Idiopathic Pulmonary Fibrosis in Routine Practice. Cells.

[66] Wang H, Zhuang Y, Peng H, Cao M, Li Y, Xu Q (2019). The relationship between MUC5B promoter, TERT polymorphisms and telomere lengths with radiographic extent and survival in a Chinese IPF cohort. Scientific reports.

[67] Moll M, Peljto AL, Kim JS, Xu H, Debban CL, Chen X (2023). A Polygenic Risk Score for Idiopathic Pulmonary Fibrosis and Interstitial Lung Abnormalities. American journal of respiratory and critical care medicine.

[68] Karman J, Wang J, Bodea C, Cao S, Levesque MC (2021). Lung gene expression and single cell analyses reveal two subsets of idiopathic pulmonary fibrosis (IPF) patients associated with different pathogenic mechanisms. PLoS One.

[69] Doubkova M, Kriegova E, Littnerova S, Schneiderova P, Sterclova M, Bartos V (2021). DSP rs2076295 variants influence nintedanib and pirfenidone outcomes in idiopathic pulmonary fibrosis: a pilot study. Therapeutic advances in respiratory disease.

[70] Wilson AC, Chiles J, Ashish S, Chanda D, Kumar PL, Mobley JA (2022). Integrated bioinformatics analysis identifies established and novel TGFβ1-regulated genes modulated by anti-fibrotic drugs. Scientific reports.

[71] Blumhagen RZ, Humphries SM, Peljto AL, Lynch DA, Cardwell J, Bang TJ (2025). MUC5B Genotype and Other Common Variants Are Associated with Computational Imaging Features of Usual Interstitial Pneumonia. Ann Am Thorac Soc.

[72] Chambers DC, Yusen RD, Cherikh WS, Goldfarb SB, Kucheryavaya AY, Khusch K (2017). The Registry of the International Society for Heart and Lung Transplantation: Thirty-fourth Adult Lung And Heart-Lung Transplantation Report-2017; Focus Theme: Allograft ischemic time. The Journal of Heart and Lung Transplantation: The Official Publication of the International Society for Heart Transplantation.

[73] Silhan LL, Shah PD, Chambers DC, Snyder LD, Riise GC, Wagner CL (2014). Lung transplantation in telomerase mutation carriers with pulmonary fibrosis. The European respiratory journal.

[74] Snyder ME, Anderson MR, Benvenuto LJ, Sutton RM, Bondonese A, Koshy R (2023). Impact of age and telomere length on circulating T cells and rejection risk after lung transplantation for idiopathic pulmonary fibrosis. The Journal of heart and lung transplantation: the official publication of the International Society for Heart Transplantation.

[75] Hannan SJ, Iasella CJ, Sutton RM, Popescu ID, Koshy R, Burke R (2023). Lung transplant recipients with telomere-mediated pulmonary fibrosis have increased risk for hematologic complications. American journal of transplantation: official journal of the American Society of Transplantation and the American Society of Transplant Surgeons.

[76] Alder JK, Sutton RM, Iasella CJ, Nouraie M, Koshy R, Hannan SJ (2022). Lung transplantation for idiopathic pulmonary fibrosis enriches for individuals with telomere-mediated disease. The Journal of heart and lung transplantation: the official publication of the International Society for Heart Transplantation.

[77] Phillips-Houlbracq M, Mal H, Cottin V, Gauvain C, Beier F, Sicre de Fontbrune F (2022). Determinants of survival after lung transplantation in telomerase-related gene mutation carriers: A retrospective cohort. American journal of transplantation: official journal of the American Society of Transplantation and the American Society of Transplant Surgeons.

[78] Zaman T, Moua T, Vittinghoff E, Ryu JH, Collard HR, Lee JS (2020). Differences in Clinical Characteristics and Outcomes Between Men and Women With Idiopathic Pulmonary Fibrosis: A Multicenter Retrospective Cohort Study. Chest.

[79] Ley B, Ryerson CJ, Vittinghoff E, Ryu JH, Tomassetti S, Lee JS (2012). A multidimensional index and staging system for idiopathic pulmonary fibrosis. Annals of Internal Medicine.

[80] van der Vis JJ, Prasse A, Renzoni EA, Stock CJW, Caliskan C, Maher TM (2023). MUC5B rs35705950 minor allele associates with older age and better survival in idiopathic pulmonary fibrosis. Respirology (Carlton, Vic).

[81] Mota PC, Soares ML, Vasconcelos CD, Ferreira AC, Lima BA, Manduchi E (2022). Predictive value of common genetic variants in idiopathic pulmonary fibrosis survival. Journal of molecular medicine (Berlin, Germany).

[82] van der Vis JJ, Snetselaar R, Kazemier KM, ten Klooster L, Grutters JC, van Moorsel CHM (2016). Effect of Muc5b promoter polymorphism on disease predisposition and survival in idiopathic interstitial pneumonias. Respirology (Carlton, Vic).

[83] Al P, Y Z, Te F, Sf M, Jg G, Tj R (2013). Association between the MUC5B promoter polymorphism and survival in patients with idiopathic pulmonary fibrosis. JAMA.

[84] Cai S, Allen RJ, Wain LV, Dudbridge F (2023). Reassessing the association of MUC5B with survival in idiopathic pulmonary fibrosis. Ann Hum Genet.

[85] Stuart BD, Lee JS, Kozlitina J, Noth I, Devine MS, Glazer CS (2014). Effect of telomere length on survival in patients with idiopathic pulmonary fibrosis: an observational cohort study with independent validation. The Lancet Respiratory medicine.

[86] Zhang D, Povysil G, Newton CA, Maher TM, Molyneaux PL, Noth I (2022). Genome-wide Enrichment of TERT Rare Variants in Idiopathic Pulmonary Fibrosis Patients of Latino Ancestry. American journal of respiratory and critical care medicine.

[87] Saito S, Lasky JA, Hagiwara K, Kondoh Y (2018). Ethnic differences in idiopathic pulmonary fibrosis: The Japanese perspective. Respiratory investigation.

[88] Clynick B, Corte TJ, Jo HE, Stewart I, Glaspole IN, Grainge C (2022). Biomarker signatures for progressive idiopathic pulmonary fibrosis. The European respiratory journal.

[89] Yeo HJ, Ha M, Shin DH, Lee HR, Kim YH, Cho WH (2024). Development of a Novel Biomarker for the Progression of Idiopathic Pulmonary Fibrosis. International journal of molecular sciences.

[90] Son JY, Kim SY, Cho SH, Shim HS, Jung JY, Kim EY (2013). TGF-β1 T869C polymorphism may affect susceptibility to idiopathic pulmonary fibrosis and disease severity. Lung.

[91] Xaubet A, Marin-Arguedas A, Lario S, Ancochea J, Morell F, Ruiz-Manzano J (2003). Transforming growth factor-beta1 gene polymorphisms are associated with disease progression in idiopathic pulmonary fibrosis. Am J Respir Crit Care Med.

[92] Liu B, Zhang X, Liu Z, Pan H, Yang H, Wu Q (2024). A novel model for predicting prognosis in patients with idiopathic pulmonary fibrosis based on endoplasmic reticulum stress-related genes. Cell biology international.

[93] Bao Y, Yang S, Zhao H, Wang Y, Li K, Liu X (2024). A prognostic model of idiopathic pulmonary fibrosis constructed based on macrophage and mitochondria-related genes. BMC pulmonary medicine.

[94] Huang T, Zhou H-F (2022). A Novel 5-Methylcytosine- and Immune-Related Prognostic Signature Is a Potential Marker of Idiopathic Pulmonary Fibrosis. Computational and mathematical methods in medicine.

[95] Lasky JA, Case A, Unterman A, Kreuter M, Scholand MB, Chaudhary S (2022). The Impact of the Envisia Genomic Classifier in the Diagnosis and Management of Patients with Idiopathic Pulmonary Fibrosis. Annals of the American Thoracic Society.

[96] Wang Y, Yella JK, Ghandikota S, Cherukuri TC, Ediga HH, Madala SK (2020). Pan-transcriptome-based candidate therapeutic discovery for idiopathic pulmonary fibrosis. Therapeutic advances in respiratory disease.

[97] Han S, Lee JE, Kang S, So M, Jin H, Lee JH (2024). Standigm ASK™: knowledge graph and artificial intelligence platform applied to target discovery in idiopathic pulmonary fibrosis. Briefings in bioinformatics.

[98] Kumazoe M, Ogawa F, Hikida A, Shimada Y, Yoshitomi R, Watanabe R (2023). Plant miRNA osa-miR172d-5p suppressed lung fibrosis by targeting Tab1. Scientific reports.

[99] Piñeiro-Hermida S, Autilio C, Martínez P, Bosch F, Pérez-Gil J, Blasco MA (2020). Telomerase treatment prevents lung profibrotic pathologies associated with physiological aging. The Journal of cell biology.

[100] Zhang S, Tong X, Liu S, Huang J, Zhang L, Zhang T (2023). AAV9-Tspyl2 gene therapy retards bleomycin-induced pulmonary fibrosis by modulating downstream TGF-β signaling in mice. Cell death & disease.

[101] Ligresti G, Caporarello N, Meridew JA, Jones DL, Tan Q, Choi KM (2019). CBX5/G9a/H3K9me-mediated gene repression is essential to fibroblast activation during lung fibrosis. JCI insight.

[102] Tan J, Tedrow JR, Dutta JA, Juan-Guardela B, Nouraie M, Chu Y (2016). Expression of RXFP1 Is Decreased in Idiopathic Pulmonary Fibrosis. Implications for Relaxin-based Therapies. American journal of respiratory and critical care medicine.

[103] Bahudhanapati H, Tan J, Dutta JA, Strock SB, Sembrat J, Àlvarez D (2019). MicroRNA-144-3p targets relaxin/insulin-like family peptide receptor 1 (RXFP1) expression in lung fibroblasts from patients with idiopathic pulmonary fibrosis. The Journal of biological chemistry.

[104] Hou J, Yang Y, Han X (2023). Machine Learning and Single-Cell Analysis Identify Molecular Features of IPF-Associated Fibroblast Subtypes and Their Implications on IPF Prognosis. International journal of molecular sciences.

[105] Cui F, Sun Y, Xie J, Li D, Wu M, Song L (2023). Air pollutants, genetic susceptibility and risk of incident idiopathic pulmonary fibrosis. The European respiratory journal.

[106] van der Vis JJ, van der Smagt JJ, Hennekam FAM, Grutters JC, van Moorsel CHM (2020). Pulmonary Fibrosis and a TERT Founder Mutation With a Latency Period of 300 Years. Chest.

[107] Tomos I, Karakatsani A, Manali ED, Kottaridi C, Spathis A, Argentos S (2022). Telomere length across different UIP fibrotic-Interstitial Lung Diseases: a prospective Greek case-control study. Pulmonology.

[108] Arai N, Nakajima M, Matsuyama M, Matsumura S, Yazaki K, Sakai C (2023). Variations in S100A8/A12 Gene Expression Are Associated with the Efficacy of Nintedanib and Acute Exacerbation Development in Idiopathic Pulmonary Fibrosis Patients. American journal of respiratory cell and molecular biology.

[109] Zhu B, Chen C, Xue G, Moyzis RK, Dong Q, Chen C (2014). The SEMA5A gene is associated with hippocampal volume, and their interaction is associated with performance on Raven's Progressive Matrices. Neuroimage.

[110] Maguire AM, Russell S, Wellman JA, Chung DC, Yu Z-F, Tillman A (2019). Efficacy, Safety, and Durability of Voretigene Neparvovec-rzyl in RPE65 Mutation-Associated Inherited Retinal Dystrophy: Results of Phase 1 and 3 Trials. Ophthalmology.

